# Identification of informative features for predicting proinflammatory potentials of engine exhausts

**DOI:** 10.1186/s12938-017-0355-6

**Published:** 2017-08-18

**Authors:** Chia-Chi Wang, Ying-Chi Lin, Yuan-Chung Lin, Syu-Ruei Jhang, Chun-Wei Tung

**Affiliations:** 1 School of Pharmacy, Kaohsiung Medical University, Kaohsiung, Taiwan; 2 Ph.D. Program in Toxicology, Kaohsiung Medical University, Kaohsiung, Taiwan; 3 Institute of Environmental Engineering, National Sun Yat-sen University, Kaohsiung, Taiwan; 4 National Institute of Environmental Health Sciences, National Health Research Institutes, Miaoli County, Taiwan

## Abstract

**Background:**

The immunotoxicity of engine exhausts is of high concern to human health due to the increasing prevalence of immune-related diseases. However, the evaluation of immunotoxicity of engine exhausts is currently based on expensive and time-consuming experiments. It is desirable to develop efficient methods for immunotoxicity assessment.

**Methods:**

To accelerate the development of safe alternative fuels, this study proposed a computational method for identifying informative features for predicting proinflammatory potentials of engine exhausts. A principal component regression (PCR) algorithm was applied to develop prediction models. The informative features were identified by a sequential backward feature elimination (SBFE) algorithm.

**Results:**

A total of 19 informative chemical and biological features were successfully identified by SBFE algorithm. The informative features were utilized to develop a computational method named FS-CBM for predicting proinflammatory potentials of engine exhausts. FS-CBM model achieved a high performance with correlation coefficient values of 0.997 and 0.943 obtained from training and independent test sets, respectively.

**Conclusions:**

The FS-CBM model was developed for predicting proinflammatory potentials of engine exhausts with a large improvement on prediction performance compared with our previous CBM model. The proposed method could be further applied to construct models for bioactivities of mixtures.

## Background

Engine exhausts are known to cause adverse health effects [1, 2]. Toxicities associated with the exposure of engine exhausts include carcinogenicity, mutagenicity and immunotoxicity [3–5]. Toxicity assessments of engine exhausts is essential for developing safe alternative fuels with lower toxicity [6]. Among the chemicals from engine exhausts, polycyclic aromatic hydrocarbons (PAHs) and nitro-PAHs are substances of major concern due to their known genotoxicity effects [6] and are suspected carcinogens in humans [7].

For toxicity evaluation of engine exhausts, several polycyclic aromatic hydrocarbons (PAHs) are quantified. Subsequently, a toxicity equivalence factor (TEF) method proposed by U.S. Environmental Protection Agency (EPA) is applied to estimate the overall toxicity posed by PAH mixtures. Finally, a TEQ value representing the overall toxicity of a PAH mixture is calculated based on the summation of equipotent concentrations of BaP converted from PAHs. The TEF/TEQ method has been widely applied to calculate the carcinogenicity and mutagenicity of PAH mixtures in environmental samples [8–13]. Despite its wide use, TEQ values are calculated in a simple additive manner assuming no antagonistic and synergistic effects among the chemicals and based on only routinely tested PAHs without incorporating the effects from other chemicals that could limit the usefulness of the TEF method [14]. As a complement to TEF method, Ames test [15], which is capable of determining the genotoxicity of engine exhausts, is routinely conducted to provide an overall genotoxicity of mixtures [16, 17].

In contrast to the evaluation of carcinogenicity and mutagenicity, there is currently no well-established method for the evaluation of immunotoxicity potentials for engine exhausts. Recently, a computational prediction model CBM based on chemical and biological features was developed to facilitate the immunotoxicity assessment of engine exhausts [14]. Due to the association between genotoxicity and immunotoxicity of PAHs [18], features collected from routinely conducted tests including quantification of PAHs, TEQ value and results from Ames test were utilized in the CBM model to predict the proinflammatory potentials of engine exhausts. The CBM model performed well with correlation coefficients of 0.972, 0.839 and 0.847 for training, cross-validation and test, respectively. The results also suggested that both chemical and biological features are required to develop an effective model [14]. Although, the effectiveness of CBM model for predicting proinflammatory potentials has been demonstrated, the importance of each feature should be further studied to provide insights into the relationship between features and proinflammatory potentials.

In this study, a sequential backward feature elimination (SBFE) algorithm was developed and applied to identify informative features for predicting proinflammatory potentials. The SBFE algorithm removes a feature with lowest contribution to the prediction performance iteratively. The feature selection process excluded five features from a total of 24 chemical and biological features. A FS-CBM model was developed by using the remaining 19 informative features selected by SBFE algorithm. By excluding unrelated features, the FS-CBM model achieved a high performance with correlation coefficient values of 0.997, 0.946 and 0.943 obtained from training, cross-validation and independent test, respectively. Compared with our previous CBM model [14], the FS-CBM model utilizing only 19 informative features provides 10% performance improvement for both cross-validation and independent test.

## Methods

### Samples of engine exhaust

A total of 16 engine exhaust samples were collected from a six-cylinder engine of Cummins B5.9-160 using various blends of diesel–hydrogen fuels in the Refining and Manufacturing Research Center for heavy-duty diesel engine operation at the Chinese Petroleum Corporation. Engine tests were completed using a Schenck GS-350 dynamometer under several loading conditions. Each collected sample was extracted in a Soxhlet extractor with a mixed solvent (n-hexane and dichloromethane 1:1 (v/v), 750 mL each) for 24 h. The extracts were then poured up into silica gel positioned under a layer of anhydrous Na_2_SO_4_ (about 1 cm high) and above a glass fiber support. The purified solution was concentrated to 1.0 mL by purging with ultra-pure nitrogen for GC/MSD analysis. The GC/MSD equipped with a capillary column (HP Ultra 2; 50 m × 0.32 mm × 0.17 μm) was calibrated with a diluted standard solution of 16 PAH compounds (PAH mixture-610 M from Supelco, USA) plus five additional individual PAHs (Merck, Germany). The concentrations of 21 PAH compounds, including naphthalene (Nap), acenaphthylene (AcPy), acenaphthene (Acp), fluorine (Flu), phenanthrene (PA), anthracene (Ant), fluoranthene (FL), pyrene (Pyr), benzo(a)anthracene (BaA), chrysene (CHR), cyclopenta(c,d)pyrene (CYC), benzo(b)fluoranthene (BbF), benzo(k)fluoranthene (BkF), benzo(e)pyrene (BeP), benzo(a)pyrene (BaP), perylene (PER), dibenzo(a,h)anthracene (DBA), benzo(b)chrycene (BbC), indeno(1,2,3,-cd)pyrene (IND), benzo(ghi)perylene (BghiP), and coronene (COR), were then determined as described in our previous studies [12, 13, 19, 20].

### Fluctuation Ames test

*Salmonella typhimurium* TA98 and TA100 were grown overnight in nutrient broth supplemented with ampicillin 25 μg/mL under constant shaking at 37 °C. The resulting cultures were used directly (TA98) or diluted 1:4 (TA100) with exposure medium (Moltox). The test samples, positive and negative controls were prepared as triplicates in 24-well plates. 10 μL of the tester was mixed with the bacterial overnight culture (50 μL) and exposure medium to a total volume of 250 μL/well and cultured for 90 min at 37 °C with 250 rpm constant shaking. After this pre-incubation, 2.5 mL of the histidine-deficient reversion indicator medium (Moltox) were added to each well. The mixtures were then transferred to 384-well plates (48 aliquots per test) and incubated for 48 h at 37 °C without agitation. A reversion due to mutation events can be detected by the color shift of the reversion indicator medium from purple to yellow, caused by the pH change of the medium due to the metabolic activity of the revertants. The number of wells containing revertants was determined by an absorption measurement at 590 nm. A sample is considered mutagenic when the number of revertant wells is significantly higher than the number of revertant wells in the negative control. In this study, the genotoxicity is represented as two proportions of the number of wells with revertants to the number of all tested wells for TA98 and TA100 tests.

### Cell culture and TNF-alpha detection

Human monocyte THP-1 cells were cultured in RPMI 1640 cell culture medium supplemented with 10% heat-inactivated fetal bovine serum (Hyclone) and 1% penicillin/streptomycin (Gibco) at 37 °C in 5% CO_2_. THP-1 cells (5 × 10^5^ cells/well) were treated with different samples in 48-well plates in the presence of lipopolysaccharide (0.5 μg/mL) for 48 h. The supernatants were collected and quantified for TNF-alpha, as an indicator of cell proinflammatory responses, by standard sandwich enzyme-linked immunosorbent assay (ELISA) as previously described [21, 22]. The TNF-alpha level in LPS group was designated as 100%, and the levels of TNF-alpha in each group was calculated according to the following formula:

$${\text{Proinflammatory potential}} = {\text{TNF-alpha}}_{\text{sample}} /{\text{TNF-alpha}}_{\text{LPS}} \times 100\% ,$$

where a value over 100% indicates induction and a value smaller than 100% indicates inhibition.

### Principal component regression (PCR)

Principal component regression (PCR) has been extensively used for the development of various predictive regression models [23–25]. The development of PCR model is based on a two-step method. First, principal component analysis (PCA) is applied to extract informative principal components accounting a given number of proportion of variance of data. Subsequently, the regression model is built from selected principal components using linear regression algorithms. The PCA procedure is based on an orthogonal transformation converting potentially correlated variables to linearly uncorrelated principal components. In this study, 95% of variance is utilized to select informative principal components from correlation matrix for developing regression. M5 algorithm is then utilized for selection of principal components for linear regression. The regression model can be regularized by a ridge parameter to avoid overfitting problems. A leave-one-out cross-validation (LOOCV) procedure is applied to determine the best ridge parameter *r* ∈ {2^1^, 2^0^, …, 2^−15^} giving the highest correlation coefficient. The implementation of the PCR methods is based on WEKA package [26].

### Sequential backward feature elimination algorithm

In this study, a wrapper-based feature selection method was developed and applied to identify informative features giving highest prediction performance. The feature selection method of sequential backward feature elimination (SBFE) algorithm is to remove features with lowest contribution to the prediction performance of PCR models iteratively. The prediction performance for each feature subset is evaluated by the PCR model using LOOCV. The sequential feature selection algorithms are simple yet powerful methods that have been successfully applied in several biological problems including pupylation sites [27], esophageal squamous cell carcinoma [28] and Ames-negative hepatocarcinogens [29].

## Results and discussion

### Identification of informative features

**Fig. 1:**
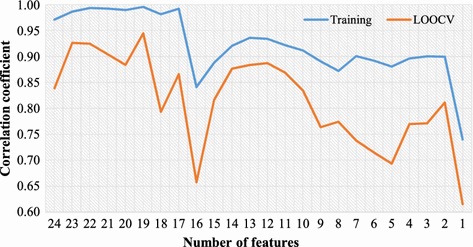
Results from feature selection

According to our previous report [14], several experiments utilizing various combinations of features have been conducted to show that both chemical and biological features are required for constructing predictive model. The results implied that most features are useful. However, the individual effects and the optimal feature subsets have not been studied. In this study, a sequential backward feature elimination (SBFE) algorithm was developed and applied to identify informative features for predicting proinflammatory potentials of engine exhausts. A total of 22 chemical features of PAHs and TEQ value and 2 biological features of TA98 and TA100 were utilized in this study. Please refer to our previous paper for a detailed dataset characteristics [14]. Figure 1 shows the results of the feature selection. For each number of features, its corresponding training and LOOCV performances were evaluated by the PCR algorithm. By removing the top five irrelevant features namely PA, BeP, PER, FL and BaP, the correlation coefficient values have been improved to 0.997 and 0.946 for training and LOOCV, respectively. The exclusion of additional features does not further improve the performance. Interestingly, the removed five features are all chemical features representing concentrations of PAHs. Among the five eliminated features, BaP is the reference PAH for the TEF method. The results might suggest that the concentration of the most carcinogenic compound BaP alone may not be highly related to proinflammatory potentials, however, the TEQ value is still useful for predicting proinflammatory potentials of engine exhausts. The final informative feature set consists of 17 chemical and 2 biological features.

### FS-CBM model

**Fig. 2:**
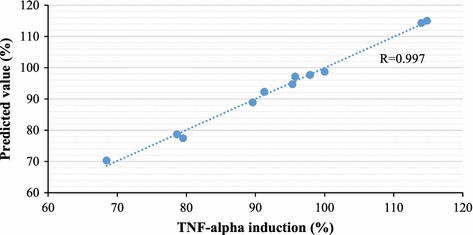
Fitting results on training set based on informative features

A final model named FS-CBM was constructed for predicting proinflammatory potentials of engine exhausts based on 11 training samples using 19 selected informative features and a ridge parameter of 2^−7^. The model gave the highest training and LOOCV performance. The fitting result is shown in Fig. 2. The FS-CBM model is shown in the following Eq. 1.

1 $$\begin{aligned} {\text{TNF-alpha}} &= - 0.6587*{\text{PC1}} + 2.319 1*{\text{PC3}} - 1 1.9916*{\text{PC5}} + 2.453 4*{\text{PC6}} \\ & \quad - 6.5432*{\text{PC7}} - 48.216 7*{\text{PC8}} + 100.3696 \end{aligned}$$

**Fig. 3:**
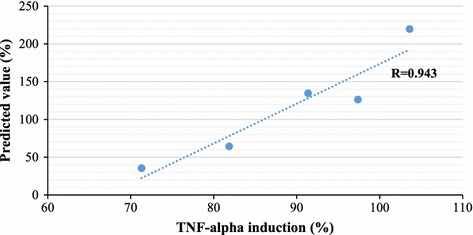
Independent test result

An independent test dataset consisting of five samples was applied to further validate the prediction performance of the developed FS-CBM model. The correlation coefficient and mean absolute error were 0.943 and 47.96, respectively. The independent test result is shown in Fig. 3. Compared with our previous study [14], there is a 10% improvement in terms of correlation coefficient on independent test dataset making the FS-CBM model more useful for predicting proinflammatory potentials of engine exhausts. The comparison of the FS-CBM model and our previous CBM model [14] is shown in Table 1. The selection of informative features provided superior performance on training, LOOCV and test.

**Table 1:** Comparison of FS-CBM and CBM model

Model	FS-CBM (this study)	CBM [14]
Number of features	19	24
Training CC	0.997	0.972
LOOCV CC	0.946	0.839
Test CC	0.943	0.849

*CC* correlation coefficient

### Feature importance

The above mentioned feature selection method successfully identified the 19 informative features for predicting proinflammatory potentials of engine exhausts. While the 19 features are essential for an effective model, the importance for each feature could provide better understanding of the relationship between features and proinflammatory potentials. Two methods applied to rank the importance of the features were shown in the follows.

The first method was to utilize the results from SBFE to provide information on the feature importance. The first eliminated feature is with lowest importance. The ranking of features according to the importance is TA98, BkF, Nap, AcPy, Acp, TEQ, COR, TA100, BbC, BaA, CYC, Flu, Pyr, BbF, CHR, Ant, BghiP, IND, DBA, BaP, FL, PER, BeP and PA. Based on the SBFE algorithm, TA98 and PA were the most important and irrelevant features, respectively. The results showed that both biological features of TA98 and TA100 are critical features for proinflammatory potentials. In summary, unlike carcinogenicity, there is no clear association between ring numbers and proinflammatory potentials as the top five important PAH features have 5, 2, 3, 3 and 6 rings. The first method provides only ranking information on relative feature importance without a quantitative measurement for their contributions to the prediction of proinflammatory potentials.

**Fig. 4:**
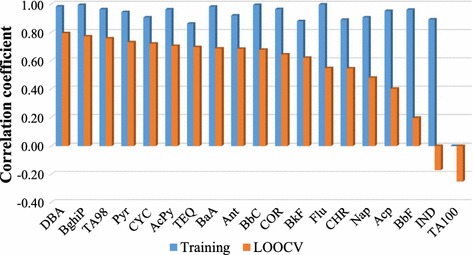
Evaluation of feature importance based on the exclusion of each feature

To give a quantitative measurement on feature importance, the second method utilized the decrease on prediction performance by excluding a specific feature that is a simple and useful method for evaluating feature importance [30]. The influence of feature exclusion on training and LOOCV performance is shown in Fig. 4. All features in the FS-CBM model were essential for an effective model according to the significant decrease on LOOCV performance. For example, the removal of TA100 feature resulted in failure to fit the training sample with the correlation coefficients of zero and negative values for training and LOOCV, respectively. By contrast, the removal of TA98 feature does not result in a failed fitting but a significant decrease on LOOCV performance. The ranking also showed no clear association between ring numbers and proinflammatory potential, which is consistent with the first method.

Altogether, biological features of TA98 and TA100 play important roles on predicting proinflammatory potentials of engine exhausts. Both methods identified Acp as one of the top ranking features indicating that the concentration of Acp could be crucial to proinflammatory potentials. Our results are consistent with previous studies in which PAHs have been reported as potent immunotoxic environmental toxicants on monocytes [18, 31, 32]. Experimental evidence suggested a direct link between carcinogenicity and immunotoxicity of PAHs [18, 31]. BaP, BaA, BbF, BkF, DBA and IND were found to induce toxic effects on macrophages [31]. Interestingly, these PAHs, except BaP, were identified as important features in this study for predicting proinflammatory potential of engine exhausts. BaP at a low concentration (1 µM) can significantly induce caspase-3 activation and cell death [31] so that BaP could induce proapoptotic effects on macrophage rather than significantly stimulate the production of proinflammatory cytokines. The proapoptotic effect of Bap might explain the exclusion of Bap feature. In addition to the important features, the eliminated features are also consistent with a study reporting the effects of PAHs on MCP-1 (monocyte chemoattractant protein-1, one of the early stage releasing chemokines during macrophage activation) production by THP-1 cells [33]. In that study, Nap and Acp, both identified as informative features, dramatically induced the production of MCP-1. In contrast, the eliminated features of PA, BaP and FL only slightly induced MCP-1 production [33]. The selection of informative features is consistent with previous studies that the informative features of Nap and Acp are correlated with proinflammatory potentials and the eliminated features of PA, BaP and FL are not the major factors associated with macrophage activation.

## Conclusions

The toxicity prediction for a single compound has been extensively studied and numerous methods have been developed such as ligand-based quantitative structure–activity relationship (QSAR) models [34, 35], chemical–protein interaction-based models [36, 37], high-content screening assay-based models [38], and interaction profile-based inference systems of CTD [39] and ChemDIS [40, 41]. However, toxicity prediction of complex mixtures is still a challenge due to the complexity of chemical interactions in mixtures. Compared with the prediction works for single compounds, only a limited number of studies for complex mixtures has been reported, for example, the prediction of mutagenicity [42]. Besides, such prediction models often just involve chemical features such as the mass spectrometry profile without incorporation of biological features. In this study, we have developed an FS-CBM model which predicts the proinflammatory potentials of engine exhausts using both chemical and biological features. The usefulness of biological features has been shown and informative features were identified for better understanding of proinflammatory potentials for engine exhausts. The successful application of the FS-CBM model demonstrates the potential application of computational methods for toxicity predictions of mixtures. More samples are being collected and analyzed to enlarge the sample size for developing a more robust model. The combination of biological and chemical features could be further applied to the prediction of various toxicities such as skin sensitization and hepatotoxicity for mixtures.
